# THERMOGRAPHY ACCURACY IN THE DIAGNOSIS OF CARPAL TUNNEL SYNDROME

**DOI:** 10.1590/1413-785220263402e283215

**Published:** 2026-05-11

**Authors:** Maria Fernanda Franco de Oliveira dos Anjos, Sérgio Murilo Georgeto, Maycon Anderson Guedes Maciel Moreira, Rodrigo Antônio Carvalho Andraus

**Affiliations:** 1 Universidade Pitagoras Unopar Anhanguera, Programa de Pos-Graduacao em Ciencias da Reabilitacao, Londrina, PR, Brazil.; 2 Hospital da Santa Casa de Londrina, Departamento de Neurocirurgia, Londrina, PR, Brazil.; 3 Universidade de Sao Paulo (USP), Programa de Pos-Graduacao em Termografia, Sao Paulo, SP, Brazil.

**Keywords:** Carpal Tunnel Syndrome, Thermography, Electromyography, Síndrome do Túnel Carpal, Termografia, Eletromiografia

## Abstract

**Objective:**

To evaluate the accuracy of thermography for diagnosing carpal tunnel syndrome (CTS) compared to electroneuromyography (ENMG), considered the gold standard, in patients with an indication for surgical treatment recruited from the neurosurgery outpatient clinic of the Irmandade da Santa Casa de Londrina.

**Methods:**

This is a cross-sectional, descriptive diagnostic accuracy study, oriented by the Guideline for Reporting Reliability and Agreement Studies (GRAAS), with a sample from the database of the project "Clinical-functional characterization and evaluation of the treatment efficacy of patients with carpal tunnel syndrome" of 44 patients, both genders, divided into a study group with 17 participants and a control group with 27 participants. A FLIR® T540 camera was used to capture the thermographic images. The data was acquired and analyzed using FLIR Tools+ software, which measured the average temperature of the region of interest. Artificial Neural Networks were used to classify the thermographic images.

**Results:**

In terms of the area under the curve was 0.908, the precision obtained was 90.81%, the accuracy 91.66%, the sensitivity 87.25% and the specificity 94.44%. The F1-score was 88.99%.

**Conclusion:**

The main findings, such as the sensitivity and specificity values, showed significant results indicating that thermography is effective in detecting CTS. **Level of Evidence I; Diagnostic studies – Investigating a Diagnostic Test**.

## RESUMO

**Objetivo:**

Avaliar a acurácia da termografia no diagnóstico da síndrome do túnel do carpo (STC) em comparação com a eletroneuromiografia (ENMG), considerada o padrão-ouro, em pacientes com indicação de tratamento cirúrgico recrutados no ambulatório de neurocirurgia da Irmandade da Santa Casa de Londrina.

**Métodos:**

Trata-se de um estudo transversal, descritivo, de acurácia diagnóstica, orientado pela Guideline for Reporting Reliability and Agreement Studies (GRAAS), com amostra proveniente do banco de dados do projeto "Caracterização clínico-funcional e avaliação da eficácia do tratamento de pacientes com síndrome do túnel do carpo", composta por 44 pacientes de ambos os sexos, divididos em grupo de estudo com 17 participantes e grupo controle com 27 participantes. Foi utilizada uma câmera FLIR® T540 para captura das imagens termográficas. Os dados foram adquiridos e analisados por meio do software FLIR Tools+, que mediu a temperatura média da região de interesse. Redes neurais artificiais foram utilizadas para classificar as imagens termográficas.

**Resultados:**

A área sob a curva foi de 0,908; a precisão obtida foi de 90,81%, a acurácia de 91,66%, a sensibilidade de 87,25% e a especificidade de 94,44%. O escore F1 foi de 88,99%.

**Conclusão:**

Os principais achados, como os valores de sensibilidade e especificidade, apresentaram resultados significativos, indicando que a termografia é eficaz na detecção da STC. **Nível de Evidência I; Estudos diagnósticos – Investigação de um exame para diagnóstico**.

## INTRODUCTION

Carpal tunnel syndrome (CTS) can involve the dominant hand, the non-dominant hand or both ^1^. The CTS is a restricted, elliptical space, ventrally confined by the flexor retinaculum; it is inelastic and resistant; its floor is formed medially by the pisiform and the hook of the hamate and laterally by the tubercle of the scaphoid and the tubercle of the trapezius ^2^.

CTS is primarily diagnosed through clinical history, physical examination, and supplementary tests, specifically neuroconduction studies CTS is diagnosed eminently clinically, through clinical history, physical examination and supplementary tests, specifically neuroconduction studies^3^. Loss of discrimination between two points in the distribution of the median nerve and tenar atrophy occur in the later stages and show low sensitivity and high specificityLoss of discrimination between two points in the distribution of the median nerve and tenar atrophy occur in the later stages of CTS.

While diagnostic imaging is not routinely required for initial diagnosis in most cases^4^, it can be valuable in specific situations, such as dubious cases, recurrent or unrelieved symptoms after surgical release of the carpal tunnel, and importantly, for evaluating surrounding soft tissue structures in the carpal tunnel. Ultrasound and MRI, for example, allow direct visualization of median nerve compression and these other tissues^5^.

Although electroneuromyography (ENMG) is accepted as the gold standard for diagnosis, it does not provide information on the surrounding tissues, which could be important for the etiological diagnosis^6^.

ENMG alone is unable to confirm or rule out the diagnosis of CTS, but it is useful for characterizing the severity of median nerve neuropathy and aiding in differential diagnosis^7^. In recent years, the use of thermography to help diagnose inflammatory conditions has been questioned. Thermographic images show temperature changes in certain body regions, helping with diseases diagnosis and treatment^8^.

Therefore, the internationally standardized cutaneous thermometric evaluation is carried out by always comparing the corresponding halves (dimidia) of the human body^9^. Infrared thermography is the preferred method for studying the physiology and thermoregulation of thermal dysfunction associated with pain^10,11^.^.^

Current image acquisition systems consist of sophisticated thermal cameras coupled to computers with specific programs where images can be processed to obtain reliable information, making thermography a safe and accurate diagnostic method^12^.

The problem with CTS is that it can be misdiagnosed and other syndromes may be diagnosed as CTS. A study of nerve conduction and temperature distribution may prove beneficial in clarifying the diagnosis^13^.

The aim of the study is to evaluate the accuracy of surface thermography and compare it with ENMG, considered the gold standard in the CTS diagnosis, in patients with an indication of surgical treatment recruited from the neurosurgery outpatient clinic of the Irmandade da Santa Casa de Londrina.

The evaluation of this technological tool as a safe and reliable diagnostic resource could contribute to new knowledge in the process of investigating CTS and lead to better treatment results, as well as being a technique with low operating costs, avoiding the immediate use of more expensive tests and lower costs for public authorities, generating a significant scientific and social contribution.

## MATERIALS AND METHODS

### Type of Study

This is a cross-sectional, descriptive diagnostic accuracy study, oriented by the Guideline for Reporting Reliability and Agreement Studies (GRAAS)^14^, carried out at *Universidade Pitágoras Unopar*. The data analyzed in this study was collected in Londrina-PR.

### Ethical Procedures

The study was approved by the ethics committee of the Irmandade da Santa Casa de Londrina under number 3.276.439, and the Informed Consent Form was signed by all participants, after an induction session where a member of the team provided a verbal description of all the measures and procedures to be carried out.

### Eligibility criteria

Inclusion criteria - age 18 or over, having idiopathic bilateral CTS, presenting one or more of the clinical criteria defined by the American Academy of Orthopaedic Surgeons, normal laboratory tests to exclude associated pathology (blood count, renal function, glycemic curve, rheumatic profile and thyroid profile), having ENMG criteria indicating mild or moderate impairment in the different combinations between the hands, and no upper limb limitations or skin lesions preventing them from undergoing the proposed therapies.

Exclusion criteria - participants with a background of psychiatric disorders or intellectual disability, pregnant, who had received previous treatment for CTS or who had been symptomatic for a period of no more than six months.

### Study Sample

This study sample was made up of 44 participants of both genders, 17 of whom were recruited from the neurosurgery outpatient clinic of the *Irmandade da Santa Casa de Londrina* with idiopathic carpal tunnel syndrome with indication of surgical treatment, while the other 27 individuals formed the control group. All the participants in the sample underwent ENMG and thermography tests.

Participants selected for the present study were included based on having idiopathic carpal tunnel syndrome with a clear indication for surgical treatment. Specifically, eligibility for surgical intervention was determined by criteria including the presence of idiopathic bilateral CTS and electroneuromyography (ENMG) indicating mild or moderate impairment in one of the hands, according to Stevens’ (1997) criteria.

The statistical program SPSS (Statistical Package for Social Sciences version 22) was used to analyze the data in which frequencies, means and standard deviations were calculated for each variable.

Anthropometric data was assessed for normality using the Shapiro-Wilk test. For normal data, the difference between the study and control groups was checked using the student's t-test for independent samples and, for data with no normal distribution, the Mann-Whitney test. The chi-square test (Fisher's exact test) was used for categorical data. A 95% confidence interval and 5% significance level (p<0.05) were used for all tests.

### Study Development

**Figure 1:**
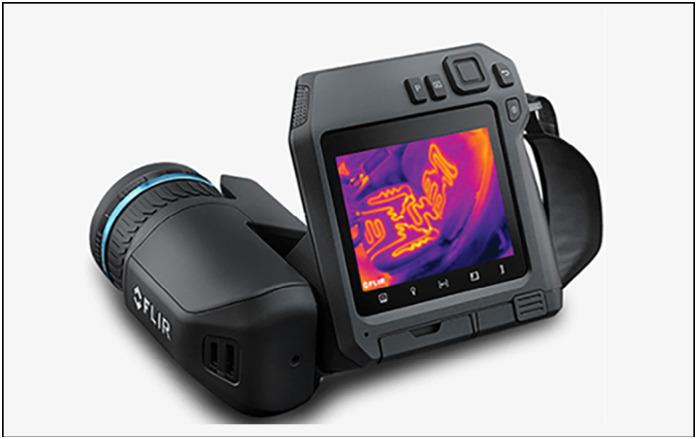
Thermographic imaging equipment (Camera FLIR® T540).

ENMG was used to capture neuromuscular activity and a FLIR® T540 camera was used to record thermographic images (Figure 1).

The patients remained in an orthostatic position for 20 minutes to become acclimatized before commencing the measurements. This position was chosen to achieve balance in skin temperature and the patients positioned in an elongated state.

All measurements were carried out in a room with controlled environment, temperature set at 22 ± 2°C. The data was acquired and analyzed using FLIR Tools+ software, which assessed the average temperature of the region of interest (ROI). This average ROI temperature was obtained from both upper limbs separately and the maximum and minimum temperatures were analyzed using the software to obtain the average temperature.

Artificial Neural Networks (ANN) were used to classify the thermographic images. The MobileNetV2 architecture is a convolutional neural network (CNN)24. The first processing layer consists of 32 convolutional filters. Generating a 32-channel activation map. The next 19 layers are residual bottlenecks. A bottleneck layer consists of 3 convolution operations.

The first is a 1x1 convolution, which means that the operation only takes into account the different channels of a single point. The second layer is a two-dimensional convolution in a 3x3 neighborhood, applied to each channel separately. This step has a non-linear activation, i.e., the result of the convolution is transformed using an activation function. The last operation is another 1x1 convolution.

**Figure 2:**
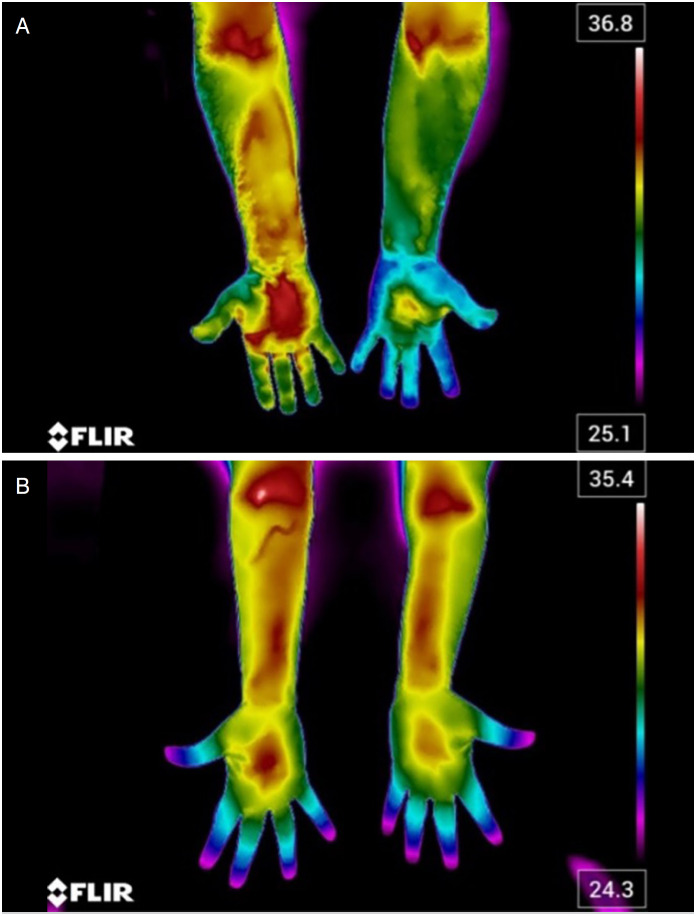
Thermographic images of the wrists of individuals in the study group (A) showing bilateral asymmetric temperature distribution and the control group (B) showing bilateral symmetric temperature distribution.

In the case of a binary classification, this layer has only 1 node. For the classification, images of affected patients were labeled 1 and images of normal patients were labeled 0. Figure 2 shows thermographic images of both the control group and the study group.

### Convolutional neural network (CNN) performance

The initial data set consisted of 27 images of regular cases and 17 images of carpal tunnel syndrome cases, all captured by the same operator using a FLIR T530 camera with 320x240 resolution. In order to carry out a more thorough validation of A.I. efficiency, we artificially increased the image bank by performing two operations.

1. For each image in the image bank, a new mirror image was created, doubling the number of elements in the analysis bank.
2. Next, two new versions for each image were created, changing the temperature of all the pixels in the image. To one image 0.5°C was added and to the Other one 0.5°C was subtracted.

In this way, we will have 5 new images for each image in the original database. By performing the two operations, we obtained a total of 162 regular cases and 102 cases of patients diagnosed with carpal tunnel syndrome. Although the operation of varying the temperature in each pixel changes the nature of the image, it does help A.I. not to solely base its analysis on the temperature of the pixel, but rather on the temperature variations throughout the human body.

This process helps AI to focus on the temperature distribution pattern of the healthy body and mitigates the influence of factors that can globally alter the temperature value, such as lack of calibration in the sensors, room temperature variation during collection, altered metabolism due to coffee intake. The ML.NET library was used to train and test the network.

The architecture used for the classifier was MobileNetV225. Cross-validation with 5 different groups was used to obtain the following statistics. Each group was used as a test set for a model trained with the other 4 groups. In order to carry out the cross-validation, the images were split before the operations to increase the database. Therefore, if an image is in the training set, none of its 5 variants will be in the test set.

Performance measures were calculated by considering the cases involving people diagnosed with carpal tunnel syndrome as positive, those where the neural network output was > 0.5. An ROC curve was generated, as well as the standard metrics, sensitivity and specificity.

## RESULTS

The study included 44 individuals of both genders with an average age of (49±11) years. Descriptions of the anthropometric data are shown in Table 1. According to the data found in the comparisons between the individuals in the study group and the control group, the variables gender, height and weight did not show any statistically significant difference, only the variable age showed a difference.

**Table 1:** Comparisons of anthropometric characteristics between individuals in the study group and the control group.

Variable	Study Group (n=17)	Control Group (n=27)	p
Sex, M/F (%)	2(12)/15(88)	1(4)/26(96)	0.549
Age (years)	55.3 (± 11.8)	45.9 (± 10.0)	0.007
Height (mt)	158.5 (± 4.7)	160.4 (± 5.8)	0.267
Weight (Kg)	75[68-87]	73[66-85]	0.595

Categorical data described in absolute (relative) frequencies. M: Male, F: Female. Numerical data described as mean (± standard deviation) or median [interquartile range 25% -75%].

The following confusion matrix shows the number of occurrences of each combination between the class to which the image belongs and the class assigned by the classifier. (Table 2)

**Table 2:** Confusion matrix of two classes (Positive/Negative).

Confusion matrix		ENMG	
		Positive	Negative
Termography	Positive	89	9
	Negative	13	153

Data obtained from the ENMG and Thermography diagnostic tests of both the study and control groups.

Precision was 90.81%, accuracy 91.66% and F1-score 88.99%. Table 3 shows the results of the specificity, sensitivity, positive predictive value and negative predictive value of thermography in relation to ENMG.

**Table 3:** Sensitivity, Specificity, PPV and NPV of Thermography compared to ENMG.

	Termography X ENMG
Sensitivity	87.2%
Specificity	94.4%
PPV	90.0%
NPV	92.0%

PPV: positive predictive value; NPV: negative predictive value.

In terms of the Operating Characteristics Curve(OCC) of the trained CNN, the area under the curve (AUC) was 0.908.

## DISCUSSION

In the present study, thermography was used in patients with carpal tunnel syndrome and ENMG was used as the gold standard. Satisfactory results were obtained from the thermographic images classified by the convolutional neural network for disease detection. The sensitivity and specificity achieved were 87.25% and 94.44%, respectively, indicating that thermography is a suitable method to supplement clinical diagnosis.

CTS is the most common compressive peripheral neuropathy and is accurately diagnosed in most cases when there is an association between pain, nocturnal numbness and positive Tinel, Phalen and Durkan tests, the latter being the most sensitive for detecting CTS on physical examination^15^.

In their studies, De Jesus Filho et al^16^ confirmed that the ENMG sensitivity for diagnosing CTS was significantly higher than the USG sensitivity and the sensitivity of the three physical examination tests (Tinel, Phalen and Durkan) evaluated in isolation. In combination, the three clinical tests showed higher sensitivity than USG.

Ultrasound is a useful method for evaluating superficial musculoskeletal structures, but it does not identify minor injuries such as muscle contusions and cramps^17^. According to Carvalho et al^18^, electroneurographic evaluation would be recommended in cases of symptomatic patients with a negative ultrasound diagnosis.

According to Fowler et al^19^ US and ENMG have similar diagnostic accuracy to clinical tests, but ENMG can diagnose other etiologies of hand paresthesia besides CTS, such as cervical radiculopathy, cubital tunnel syndrome and pronator syndrome. Electroneuromyography confirms the clinical diagnosis of CTS with a high degree of sensitivity (over 85%) and specificity (over 95%)^20^.^.^

Electroneuromyography should be requested whenever neuropathy is suspected. However, it is an invasive method, sometimes uncomfortable, and the result may not show the presence of nerve compression. The test results may also show no injuries as it is not possible to detect them^21^.

Many studies have focused on thermographic wrist evaluation, especially in carpal tunnel syndrome, tendonitis and myofacial syndromes, all of which have shown statistically significant positive results correlating images with local disorders^22^.

In a study by Zyvcák, Hudäk and Madaras^23^, they also obtained satisfactory results when using thermography in patients with carpal tunnel syndrome. According to the authors, sensitivity and specificity were 71% and 82% respectively, indicating that thermography is a suitable method to complement clinical diagnosis.

Côrte and Hernandez^24^ reported that the main advantage of thermography is its safety, however, its disadvantage results from its physical limitations. The two-dimensional, non-radiating technique provides information on superficial structures.

Consequently, while thermography shows promise as a diagnostic tool and a valuable supplement to clinical examination, it cannot be considered a standalone method for the comprehensive diagnosis of CTS. This is because, unlike electroneuromyography (ENMG), which is useful for characterizing nerve severity and critically, aiding in the differential diagnosis of other conditions mimicking CTS symptoms, thermography's current application primarily focuses on thermal patterns associated with the condition itself rather than identifying alternative etiologies or providing detailed information on surrounding tissues important for etiological diagnosis.

Thermography, like many other diagnostic test, thus requires confirmation from other modalities to ensure diagnostic accuracy^9,11^ and can be used to monitor the patient's progress and treatment^25^.

Romano^26^ stated that thermography is useful for identifying signs of pathology and that there is much evidence that diseases or dysfunctions are often associated to changes in skin temperature.

Ramos et al^27^ checked the skin behavior of the wrist and finger extensors during a brief typing task in individuals with and without injuries. Lower minimum, average and maximum temperatures were observed in the elbow region in individuals with injuries. Lower temperatures were found on the right side when compared to the left side in the elbow skin region. The presence of chronic injuries leads to discontinue the use of the musculoskeletal structures, resulting in reduced blood supply and therefore lower temperatures.

Thermography is a suitable tool for assessing and preventing muscle injuries in athletes, but care must be taken with the control variables during its use. The most efficient variables for capturing the thermographic image seem to be in an environment with a temperature between 18 and 25°C, for 15 minutes for acclimatization and with the individual placed in a predetermined position, depending on the body segment that is being assessed, without contact with another object^28,29^.

Thermal imaging offers an objective criterion in supplementary diagnosis of fibromyalgia, which generally has vague symptoms associated with a psychosomatic component. These patients have a non-specific hyper-radiant pattern corresponding to the classic painful muscle areas, due to local muscle hypertonia; unlike myofascial trigger points which have a very regular, ellipsoid thermal image with well-defined contours. Infrared can be an additional diagnostic method for documenting and monitoring fibromyalgia^30^.

For Magas et al^30^ the main findings in their study refer to changes in variation rates and mean temperature difference, as well as sensitivity and specificity values, which showed meaningful results indicating that thermography can detect wrist tendonitis. The study found that thermography has high sensitivity, ranging from 71% to 100% according to the listed studies, and is increasingly gaining ground in diagnostic support, monitoring and assessment of musculoskeletal symptoms, especially in wrist, elbow and shoulder pathologies.

## CONCLUSION

Based on the findings, thermography was proven to have significant sensitivity and specificity values, it can then be concluded that thermography is a non-invasive, painless and promising method in the diagnostic process of CTS, unlike ENMG, which is an invasive and sometimes uncomfortable method.

## Data Availability

The data set for this article is available in the digital repository: https://data.mendeley.com/datasets/m7wfbxhfg3/1

## References

[1] Lima DF, Lima LA (2017). Prevalência da síndrome do túnel do carpo em trabalhadores que lidam com a ordenha manual de bovinos. Revista Dor.

[2] Machado DA, Martins WP (2009). Síndrome do túnel do carpo. EURP.

[3] Alves MPT (2010). Estudo prospectivo comparativo entre a descompressão do canal do carpo pela mini-incisão transversa proximal e a incisão palmar longitudinal convencional. Rev Bras Ortop.

[4] Katz JN, Simmons BP (2002). Clinical practice. Carpal tunnel syndrome. N Engl J Med.

[5] Kirchhoff DC, Monducci D, Alves LP, Pereira L, Okuda FAF, Takey AM (2012). Casuística e Follow Up de 1.639 casos de Síndrome do Túnel do Carpo operados por técnica aberta protocolada no serviço. Comparação dos nossos resultados com os obtidos por outras técnicas segundo a literatura. Original J Bras Neurocirurg.

[6] Alves MPT (2013). Síndrome do túnel do carpo: estudo comparativo entre as medidas ultrassonográfica e cirúrgica do nervo mediano. Radiol Bras.

[7] Figueiredo R (2018). O Papel da Ultrassonografia no acompanhamento da Síndrome do Túnel do Carpo. RBUS.

[8] Brioschi ML, Mehl A, Oliveira A, Freitas MAS, Macedo JF, Matias J (2007). Exame de termometria cutânea infravermelha na avaliação do pé diabético. Rev Méd Paraná.

[9] Brioschi ML, Macedo JF, Macedo RAC (2020). Termometria cutânea: novos conceitos. J Vasc Br.

[10] Marçal MA, Elias APV, Silva FFD (2016). Uso da termografia infravermelha na identificação de dor em trabalhadores encaminhados para reabilitação. https://pdf.blucher.com.br/engineeringproceedings/conaerg2016/8088.pdf.

[11] Brioschi ML, Macedo JF, Macedo RAC (2003). Termometria cuta?nea: novos conceitos. J Vasc Br.

[12] de Meira LF, Krueger E, Neves EB, Nohama P, de Souza MA (2014). Termografia na área biomédica. Pan Am J Med Thermol.

[13] Souza EM, Fernandes FA, Soares CLA, Seixas FL, Santos AAS, Gismondi RA (2020). Inteligência artificial em cardiologia: conceitos, ferramentas e desafios - "quem corre é o cavalo, você precisa ser o jóquei". Arq Bras Cardiol.

[14] Kottner J, Audigé L, Brorson S, Donner A, Gajewski BJ, Hróbjartsson A (2011). J Clin Epidemiol.

[15] Bastos VH (2009). Os efeitos da mobilização neural como abordagem ï sioterapêutica na síndrome do túnel do carpo. Fisioterapia Brasil.

[16] de Jesus AG, do Nascimento BF, de Carvalho Amorim M, Naus RAS, de Araújo Loures E, Moratelli L (2014). Estudo comparativo entre o exame físico, a eletroneuromiografia e a ultrassonografia no diagnóstico da síndrome do túnel do carpo. RBO.

[17] Garcia DR (2004). Validação da termografia no diagnóstico de lesões por esforços repetitivos/distúrbios osteomusculares relacionados ao trabalho.

[18] Carvalho KMD, Soriano EP, Carvalho MVD, Mendoza CC, Vidal HG, Araújo ABVL (2011). Nível de evidência e grau de recomendação dos artigos sobre a acurácia diagnóstica da ultrassonografia na síndrome do túnel do carpo. Radiol Bras.

[19] Fowler JR, Cipolli W, Hanson T (2015). A Comparison of Three Diagnostic Tests for Carpal Tunnel Syndrome Using Latent Class Analysis. J Bone Joint Surg Am.

[20] Jablecki CK, Andary MT, Floeter MK, Miller RG, Quartly CA, Vennix MJ (2002). Practice parameter: Electrodiagnostic studies in carpal tunnel syndrome [RETIRED]. Report of the American Association of Electrodiagnostic Medicine, American Academy of Neurology, and the American Academy of Physical Medicine and Rehabilitation. Neurology.

[21] Teefey SA, Middleton WD, Boyer MI (2000). Seminars in Ultrasound, CT and MRI.

[22] de Trotta J, Ulbricht L (2015). Termografia no Diagnóstico Complementar de Doenças Músculo Esqueléticas [Thermography in Complementary Diagnostic of Musculoskeletal Diseases]. PAJMT.

[23] Živcák J, Madarasz L, Hudak R (2011). Application of medical thermography in the diagnostics of carpal tunnel syndrome.

[24] Côrte ACR, Hernandez AJ (2016). Termografia médica infravermelha aplicada à medicina do esporte. Rev Bras Med Esporte.

[25] Herman C, Cetingul MP (2011). Visualização quantitativa e Detecção de Câncer de Pele Usando imagens térmicas dinâmicas.

[26] Romano CL, Romano D, Dell’Oro F, Logoluso N, Drago L (2011). Healing of surgical site after total hip and knee replacements show similar telethermographic patterns. J Orthop Traumatol.

[27] Ramos L, Bertani AL, Oltramari JD, Dhein W (2020). Thermal behavior of the skin on the wrist and finger extensor muscles during a typing task. Rev Bras Med Trab.

[28] Viegas F, Mello MT, Rodrigues SA, Costa CMA, Freitas LSN, Rodrigues EL (2020). The use of thermography and its control variables: a systematic review. Rev Bras Med Esporte.

[29] Bandeira F, Moura MAM, Souza MA, Nohama P, Neves EB (2012). Pode a termografia auxiliar no diagnóstico de lesões musculares em atletas de futebol?. Rev Bras Med Esporte.

[30] Brioschi ML, Yeng LT, Pastor EMH, Teixeira MJ (2007). Utilização da imagem infravermelha em reumatologia. Rev Bras Reumatol.

